# Artificial Neural Networks-Based Software for Measuring Heat Collection Rate and Heat Loss Coefficient of Water-in-Glass Evacuated Tube Solar Water Heaters

**DOI:** 10.1371/journal.pone.0143624

**Published:** 2015-12-01

**Authors:** Zhijian Liu, Kejun Liu, Hao Li, Xinyu Zhang, Guangya Jin, Kewei Cheng

**Affiliations:** 1 Department of Power Engineering, School of Energy, Power and Mechanical Engineering, North China Electric Power University, Baoding, Hebei, 071003, PR China; 2 College of Software Engineering, Sichuan University, Chengdu, Sichuan, 610064, PR China; 3 College of Chemistry, Sichuan University, Chengdu, Sichuan, 610064, PR China; 4 National Center for Quality Supervision and Testing of Solar Heating Systems (Beijing), China Academy of Building Research, Beijing, 100013, PR China; 5 School of Computing, Informatics, and Decision Systems Engineering (CIDSE), Ira A. Fulton Schools of Engineering, Arizona State University, Tempe, Arizona, United States of America; Argonne National Lab, UNITED STATES

## Abstract

Measurements of heat collection rate and heat loss coefficient are crucial for the evaluation of in service water-in-glass evacuated tube solar water heaters. However, conventional measurement requires expensive detection devices and undergoes a series of complicated procedures. To simplify the measurement and reduce the cost, software based on artificial neural networks for measuring heat collection rate and heat loss coefficient of water-in-glass evacuated tube solar water heaters was developed. Using multilayer feed-forward neural networks with back-propagation algorithm, we developed and tested our program on the basis of 915measuredsamples of water-in-glass evacuated tube solar water heaters. This artificial neural networks-based software program automatically obtained accurate heat collection rateand heat loss coefficient using simply "portable test instruments" acquired parameters, including tube length, number of tubes, tube center distance, heat water mass in tank, collector area, angle between tubes and ground and final temperature. Our results show that this software (on both personal computer and Android platforms) is efficient and convenient to predict the heat collection rate and heat loss coefficient due to it slow root mean square errors in prediction. The software now can be downloaded from http://t.cn/RLPKF08.

## Introduction

Currently, the boosting solar water heater market worldwide promotes the demand for advanced technologies and related products. The solar water heater has become one of the most efficient and economical ways to utilize solar energy. This system, including solar collectors, heat exchanger and solar concentrators, gathers and converts energy from solar radiation and transfers it into household water [1]. In China, the water-in-glass evacuated tube solar water heaters have been widely used because of their high energy efficiency and simple installation [2, 3].The solar energy industry has developed rapidly in China. By the end of 2012, around 2.57× 10^8^ m^2^of solar water heaters was installed in China, 90% of which was water-in-glass evacuated tube solar water heaters[4]. The market share of all-glass evacuated tube solar collectors was about 88% in 2003, while by 2009 it increased to 95%[5]. From 2001 to 2009, the production of evacuated solar tubes increased at an annual rate of 30% in China[3]. More than twenty million evacuated solar tubes were produced in 2001, and by 2009 the production increased to350 million [6].

Studies using experimental, theoretical and computational fluid dynamics (CFD) simulation[7–10]had been performed to investigate and evaluate the heat collection rate, heat loss coefficient and energy efficiency of water-in-glass evacuated tube solar water heaters. Using the ISO9459-2 standard test methods, a previous study showed that the stagnant zone could significantly affect the water-in-glass collector tubes operation[11].A new-type all-glass evacuated tubular solar air heater coupled with compound parabolic concentrator may produce large flow heated air at above 200°C[12].The study for the heat transfer and fluid flow characteristics in one single tube heat collector by computational fluid simulation indicates that the twist tape inserts reduce velocity magnitude and make the temperature field more uniform[13]. The study for the effect of the factor of water temperature in tank on the thermal performance has also shown that as water temperature in tank increases, the net energy storage in the above system declines. The difference between required tank water temperature of 40°C and 80°C was over1,000 Whm^−2^d^−1^under the condition of a solar radiation gain of 8,000 Whm^−2^ d^−1^[14].One other study indicates that the outlet temperature and thermal efficiency of a low temperature water-in-glass evacuated tube solar collector by employing the Boussinesq approximation model were closer to the experimental data than those obtained by using the variation of the proprieties with temperature model[15].By a mathematical method, it is shown that the factors, including collector forms, solar tubes size, central distance between tubes, angles of azimuth and tilt, and diffuse flat reflector employment, may influence the annual collectible radiation in the single tube daily collectible radiation level of all-glass evacuated tube solar collectors[16]. One similar study of natural hydraulic circulation flow field characteristics in water-in-glass evacuated tube solar collectors, showed that the circulation flow rate was closely linked with tank temperature in tank, aspect ratio of tube, angle of collector tilt and the input of solar radiation[17].

However, the most important coefficients of thermal performance, including heat collection rate and heat loss coefficient, are very difficult to determine because the test conditions to assess the thermal performance of solar water heaters are confined by GB/T 19141–2011 standards[18]:

1. the test period is 8 h, including 4 h before solar noon and 4 h after;
2. the daily solar irradiation shall be higher than 16 MJ/m^2^;
3. the daily average surrounding temperature shall be between 8 and 39°C;
4. the daily surrounding air speed shall be less than 4 m/s;
5. the initial temperature in the storage tank shall be 20 ± 1°C.

**Fig 1:**
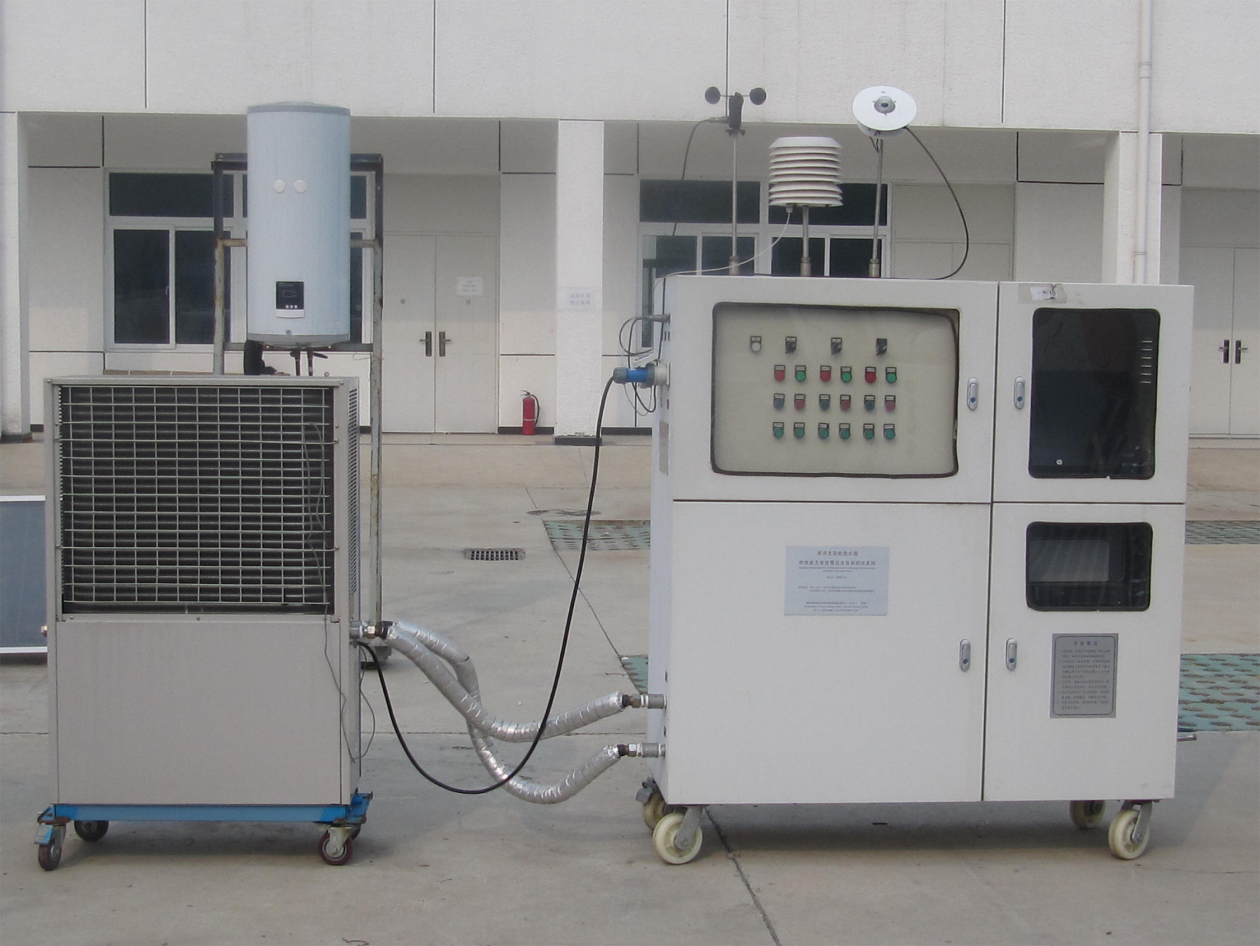
Picture of the detection deviceused to measure the heat collection rate and heatloss coefficient.

**Fig 2:**
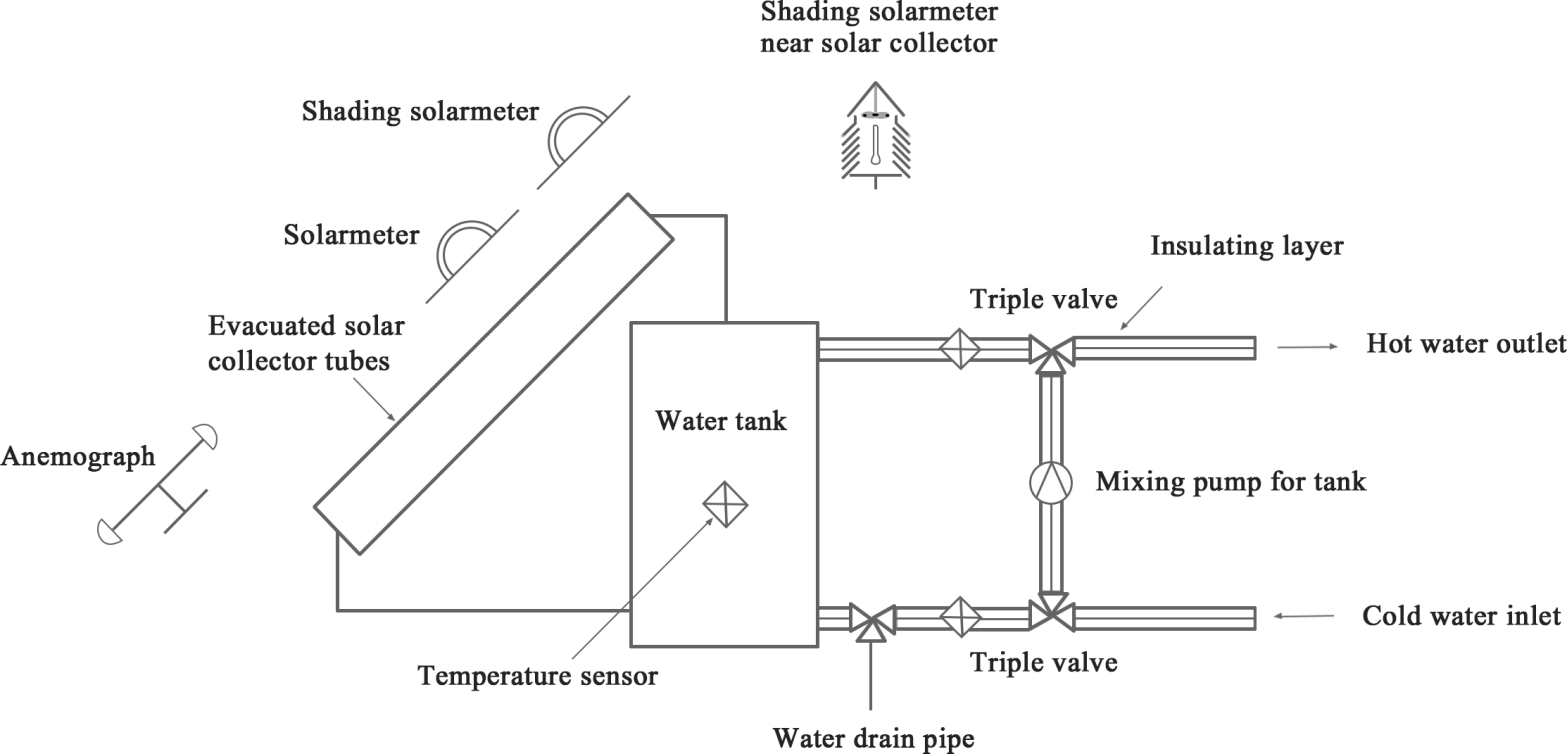
Experimental setupschematic of measuring the thermalperformance of water-in-glass evacuated tube solar water heaters.

According to the GB/T 19141–2011 standards, it requires15 days to obtain the heat collection rate of a new solar water heater in Beijing, China, which is time-consuming and strenuous[18]. Although the detection devices (PDT 2013–1), as shown in Fig 1 and the experimental setup schematic of measuring the thermal performance of water-in-glass evacuated tube solar water heaters, as shown in Fig 2, can acquire data precisely, the whole process is time-consuming and energy-consuming due to the necessary disassembly of the water heaters. In addition, this may cause damage to heaters and so far an effective solution to avoid the unnecessary damage is still unknown. Previously, we developed a novel method using some machine learning models such as support vector machine (SVM) for the prediction of heat collection rates and heat loss coefficients [19]. However, lacking of a well-developed software, users may find it difficult to use the method for practical measurements. Here, we propose a software based on artificial neural networks (ANNs) to predict the heat collection rate and heat loss coefficient without the disassembly of heaters based on experimental data. Parameters that can be precisely obtained by"portable test instruments", as shown in Table 1[19], were set as independent variables, which are all relevant to the values of heat collection rate and heat loss coefficient, including tube length(mm), number of tubes, tube center distance(mm), heat water mass in tank(kg), collector area(m^2^), angle between tubes and ground(°)and final temperature(°C).The heat collection rate (MJ/m^2^)and heat loss coefficient [(W/(Km^3^)]measured from the detection device and relevant equations were set as dependent variables. Multilayer feed-forward neural networks (MLFNs) trained with back-propagation (BP)algorithm were used for training the predictive models based on the data from 915 solar water heaters. To choose the best model for the software, comparisons were made among the models with different number of training epochs, hidden nodes, learning rates and momentums.

**Table 1:** "Portable test instruments" for the determination of parameters of water-in-glass evacuated tube solar water heaters[19].

Parameters	Portable test instruments	Accuracy
*Final temperature of water*	Digital thermoelectric thermometer	0.5%
*Hot water mass in tank*	Electric platform scale	1.0%
*Diameter*, *tube center distance*, *tube length*, *collector area*	Taper ZSH-3	0.5%

## Materials and Methods

### Experiment

According to the methods for independent and dependent variable determination in this study, 915 water-in-glass evacuated tube solar water heaters (in service for one year) were precisely obtained by the "portable test instruments" and the PDT2013-1 detection device (National Center for Quality Supervision and Testing of Solar Heating Systems, Beijing).The complete data can be downloaded from http://t.cn/RLPKF08. Table 2 shows the statistical analysis of the data.

**Table 2:** Descriptive statistic of the variables for 915 samples of in service water-in-glass evacuated tube solar water heater.

Item	Tube length (mm)	Number of tube	Tube center distance (mm)	Tank volume (kg)	Collector area (m^2^)	Angle(°)	Finaltemperature (°C)	Heat collection rate (MJ/m^2^)	Heat loss coefficient [W/(m^3^K)]
*Maximum*	2200	64	151	403	8.24	85	62	11.3	13
*Minimum*	1600	5	60	70	1.27	30	46	6.7	8
*Range*	600	59	91	333	6.97	55	16	4.6	5
*Average*	1811	21	76.2	172	2.69	46	53	8.9	10
*Standard deviation*	87.80	5.80	5.11	47.00	0.73	3.89	2.00	0.48	0.77

### ANNs Model

ANNs are effective machine learning approaches with functions of estimation and approximation based on input values [20–22], which are widely used in numeric predictions and pattern recognitions. In our studies, MLFNs were chosen for model development and BP algorithm was used as the training algorithm.

#### MLFNs

**Fig 3:**
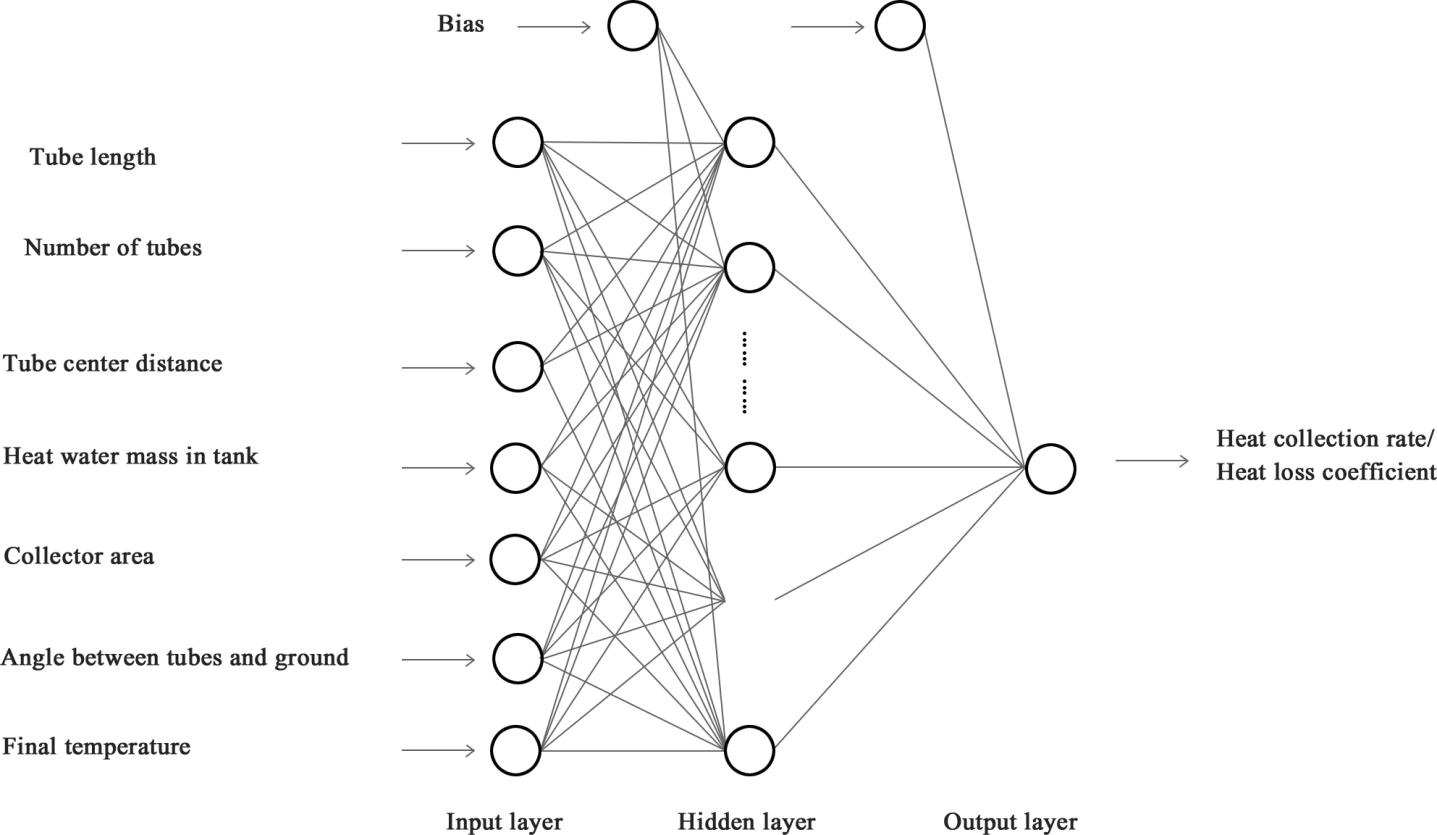
Schematic structure of an ANN in our study.

An MLFN model consists of neurons that are ordered into different layers[23–25]. The first layer is the input layer while the last layer is the output layer, and the layers between the input and output layers are the hidden layer (Fig 3). The data moves in only one direction. Each neuron in a particular layer is connected with all neurons in the next layer and each neuron is a computational unit that takes inputs, and outputs[22–24]:

$$hw,b(x)=f(\xi i)=f(\sum_{i=1}^{m}wixi+b)$$ (1)

where *ξ*_*i*_is the potential of the*i*th neuron and *f*(*ξ*_*i*_)is called the transfer function.*w*is the weight coefficient and *b*is the threshold coefficient which can be understood as a weight coefficient of the connection with formally added neuron.

#### BP Algorithm

BP algorithm adopts gradient descent as its optimization method, in which it calculates the gradient of a loss function with respects to all weights and bias in the network[26–28]. Then the gradient is fed and used to update weights and bias, in order to minimize the loss function.

In BP algorithm, sigmoid function is widely used as the transfer function[29]:

$$f(x)=\frac{1}{1+e^{-x}}$$ (2)

## Results and Discussion

### Model Development

Using the "portable test instruments", for an in service water-in-glass evacuated tube solar water heater, precise values of tube length(mm), number of tubes, tube center distance(mm), heat water mass in tank(kg), collector area(m^2^), angle between tubes and ground(°) and final temperature(°C)can be obtained easily from outdoors, whereas the heat collection rate(MJ/m^2^) and heat loss coefficient[W/(Km^3^)]can only be determined by the conventional detection device after being dismantled. To avoid complex disassembly and obtain the heat collection rate and heat loss coefficient in real time, here, we aim at using machine learning techniques, specifically, MLFNs to develop a series of prediction models for the heat collection rate and heat loss coefficient. Data acquired by "portable test instruments" were designated as independent variables, while the heat collection rate (MJ/m^2^)and heat loss coefficient [W/(Km^3^)]were designated as dependent variables. Models were developed for both heat collection rate (MJ/m^2^) and heat loss coefficient [W/(Km^3^)]in order to find out the best prediction model for each of these two dependent variables. 915 groups of completed experimental data were used for the model development. 815data groups were used as the training set, while 100 data groups were used as the testing set.

Before deciding the configuration of ANNs, factors which influence the performance of ANNs need to be analyzed. The values of weight and bias between layers in neural net, which are key to the performance of ANNs, are determined by data pre-processing, activation function, the number of training epochs, the number of nodes in hidden layer, learning rate, and the momentum. Data pre-processing is the process of reducing data to its canonical form in order to prevent certain feature with much bigger values than others from overshadowing other features. We adopt data normalization, specifically simple rescaling method to do data pre-processing here. In ANNs, the transfer function of a node defines the output of that node given an input or set of inputs. We uses igmoid function as the transfer function, between input layer, hidden layer and output layer. The final data vectors lie in the range [0.1,0.9].The number of training epochs determines the number of epochs the ANNs trained. Another important factor is the momentum, which is a useful technique that can help the network out of local minima. Thus, besides data pre-processing method and transfer function, the performance of ANNs is mainly influenced by training epochs, hidden nodes, learning rate and momentum.

In order to find out the best ANNs configuration, control variable method is adopted here. Different training epoch, hidden node, learning rate and momentum were set individually. To avoid missing the best configuration figure set, the experimental range of each parameter is as large as possible. The range of training epoch we set here is from 1 to 500. As the range of learning rate and momentum lies in [0.1, 0.9] themselves, they are set from 0.1 to 0.9. The hidden nodes of models were numbered from 1 to 13, from which we could track the change regulation of the ANNs when dealing with the development processes.

Root mean square error (RMS error) in testing and the required training time were used as indicators to measure the performance of the MLFNs. The RMS errors of each model were calculated by Eq (3):

$$RMSE=\sqrt{\frac{\sum_{i-1}^{n}{\left(Z_{i}-O_{i}\right)}^{2}}{n}}$$ (3)

Where *Z*_*i*_ is the predicted value, *O*_*i*_ is the actual value and *n* is the number of tested samples.

A runnable ANN program is written. Each time we change one particular variable in the ANN configuration figure set and run the ANN program, then the corresponding RMS errors are recorded, from which we can find the best ANNs models for heat collection rates and heat loss coefficient separately by choosing the ANNs model with the lowest RMS error.

Experiment shows that when predicting the heat collection rates, the RMS errors of the presented models vary from 0.6254 to 0.2049. The main reason for the various RMS errors is that there may be some under-fitted and over-fitted results in some configurations after trainings, leading to relatively higher RMS errors. The lowest RMS error exists in the BP with 200 epochs, 7 nodes, 0.9 learning rate, and 0.9 momentum(0.2049). Therefore, for the prediction of heat collection rate, it is reasonable to consider that the BP model with 200 epochs, 7 nodes, 0.9 learning rate and 0.9 momentum is one of the best models. Detailed results of prediction models for heat collection rate are included in S1 Table.

In terms of the prediction of heat loss coefficient, RMS errors are quite similar in different models, which vary from 1.1304to 0.9246. The reason for the variety of RMS errors is also the under-fitted and over-fitted results, which is the same as the heat collection rate models discussed above. The lowest RMS error exists in the BP with 50 epochs, 1 node, 0.1 learning rate, and 0.1 momentum (0.9246). Therefore, for the prediction of heat loss coefficient, it is reasonable to consider that the BP model with 50 epochs, 1 node, 0.1 learning rate and 0.1 momentum is one of the best models. Detailed results of prediction models for heat loss coefficient are included in S2 Table.

### Model Analysis

**Fig 4:**
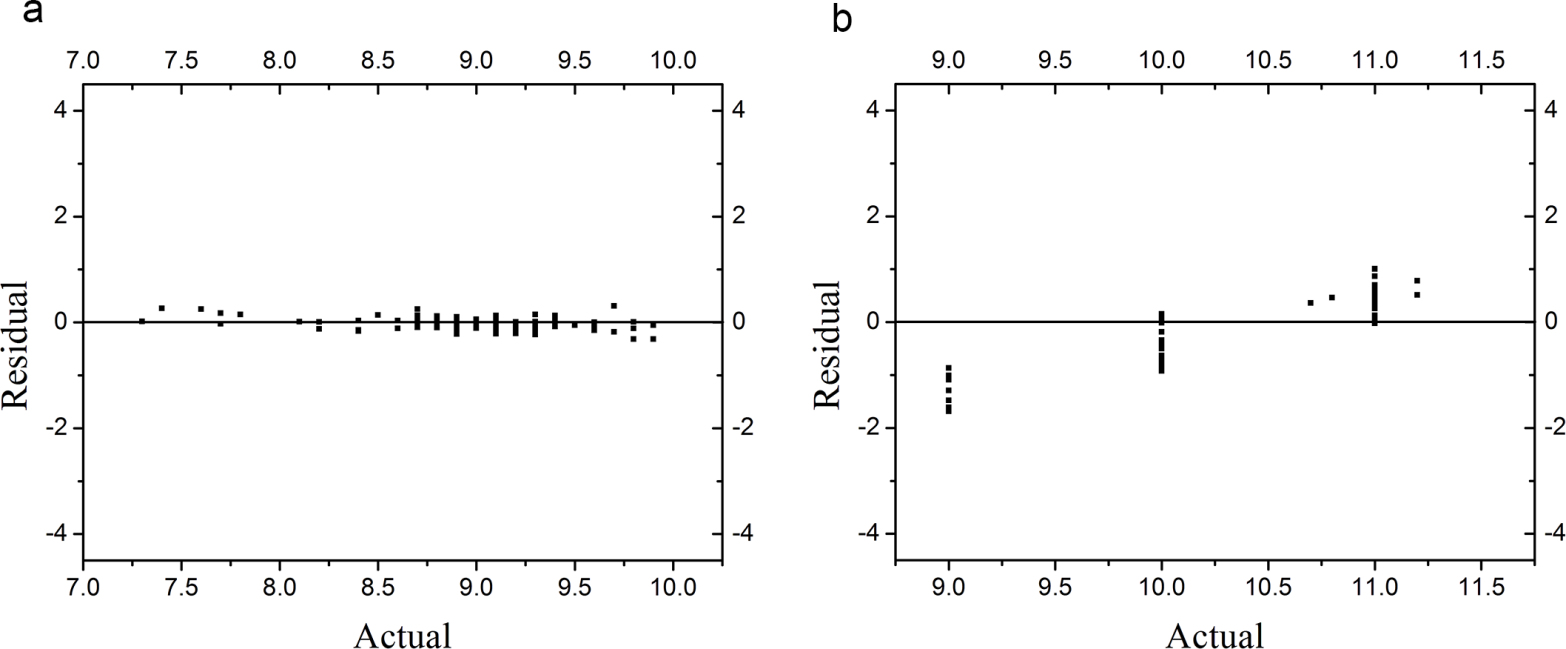
Residual values versus actual values in predicting. (a) Heat collection rate. (b) Heat loss coefficient.

Before the development of the packed software the model should be evaluated first. Fig 3 shows the comparisons between the residual values and their actual values. Residual values were calculated by Eq (4):

z=y−x (4)

where *z* is the residual value, *y* is the actual value and *x* is the predicted value.It can be seen that the prediction of heat collection rate is precise because the residual values are close to zero [Fig 4 (a)]. In terms of the prediction of heat loss coefficient, despite deviations exist when the actual values are close to 9.0, most residuals are close to zero [Fig 4 (b)]. Our results suggest that the two BP models we chose for predicting heat collection rate and heat loss coefficient respectively are applicable in actual situations.

### Software Development

**Fig 5:**
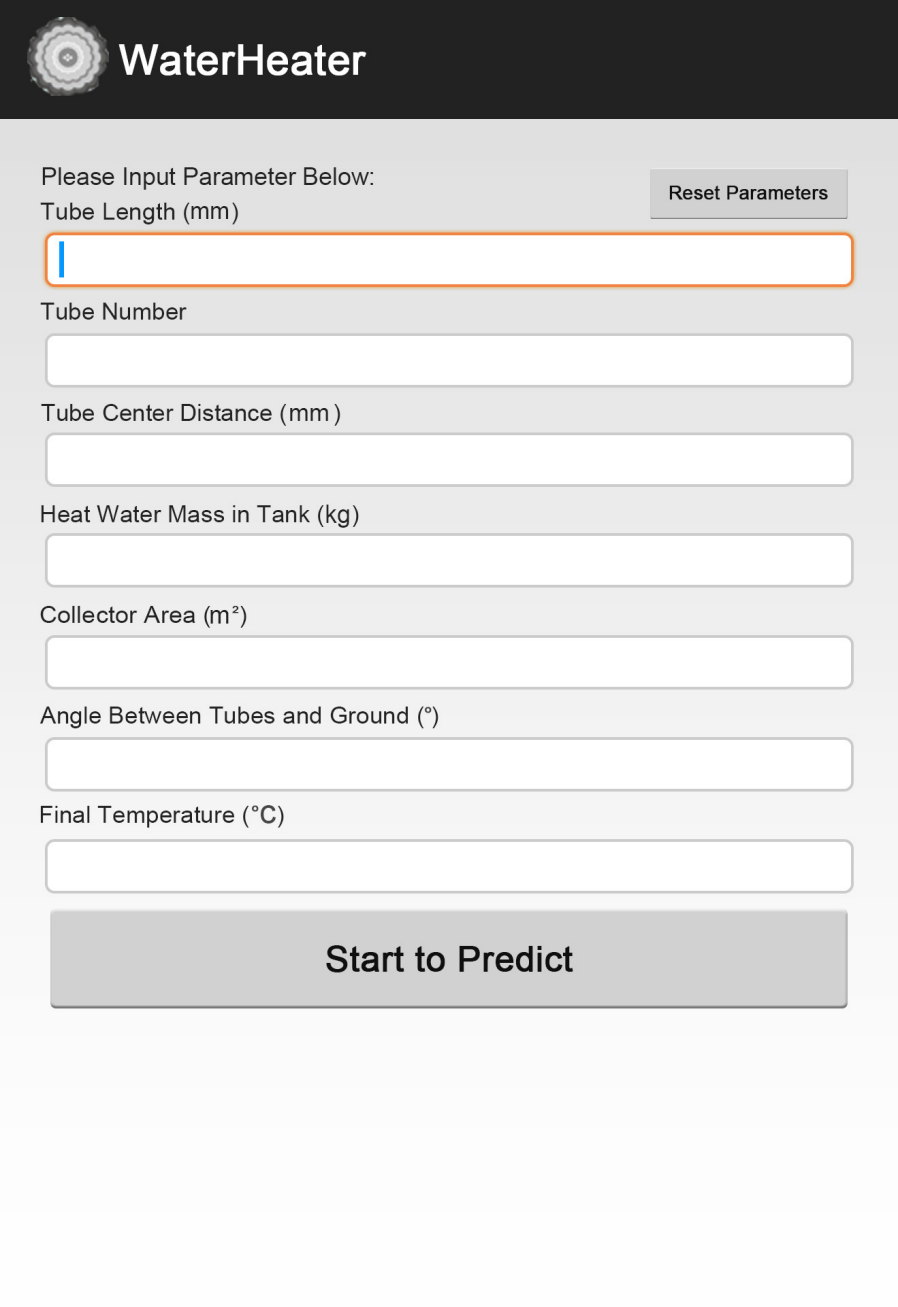
Overview panel of Android platform.

**Fig 6:**
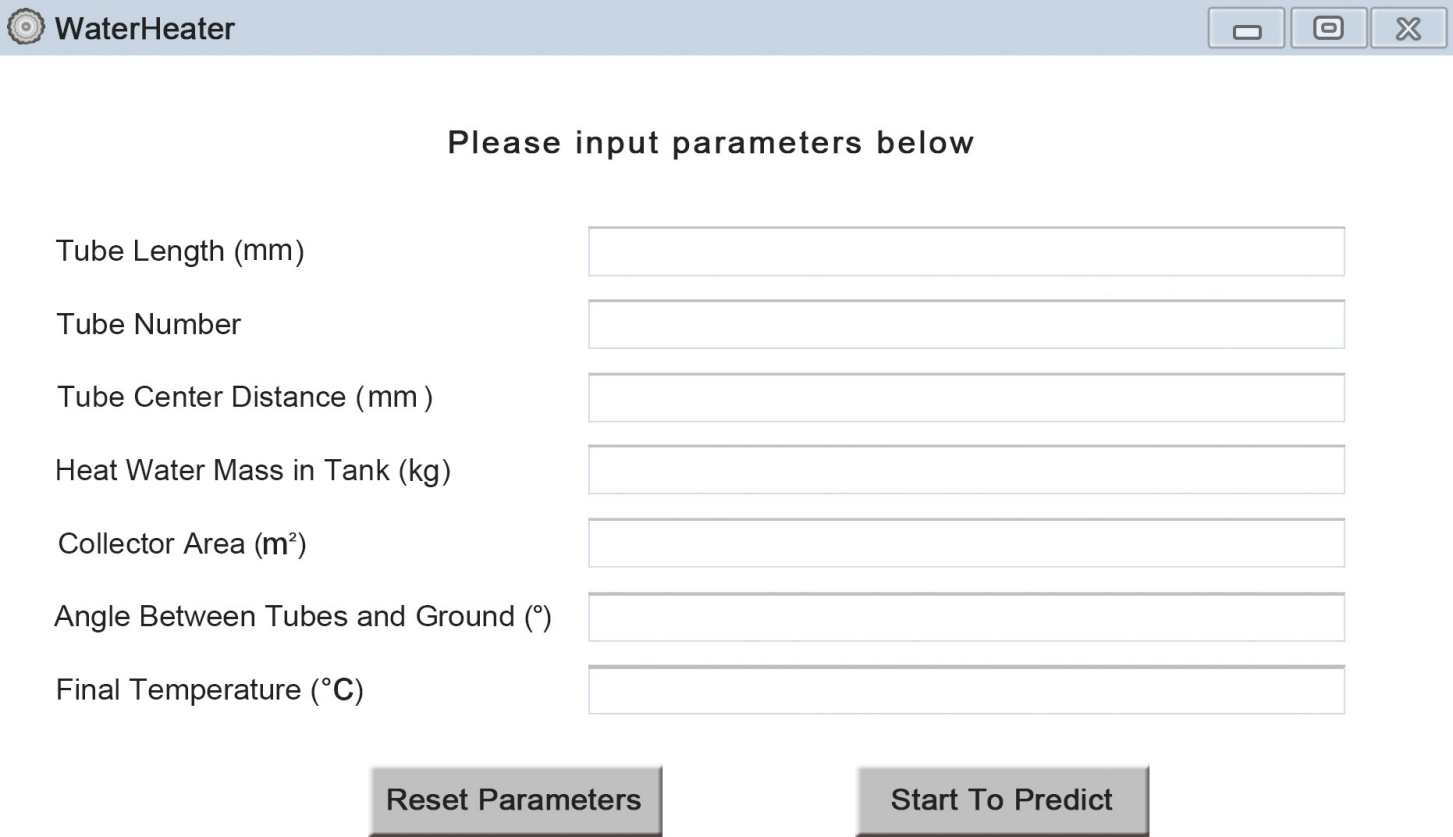
Overview panel of PC platform.

In order to offer great convenience for users work either in office with personal computer or outdoor with portable smartphone, two pieces of individual software are developed, including personal computer (PC) platform version and smartphone platform version. Considering that Android platform is now the world’s most commonly used smartphone platform (dominated the global smartphone market with a share of 82.8% in 2015)[30], in terms of smartphone platform, we developed Android platform in particular. So users are free to adopt the PC version when dealing with analysis on computer, and can also use the Android version simply with their phone when computer is not available. Figs 5 and 6 show the overview panel of our software, *WaterHeater*. The application ranges of tube length (mm), number of tubes, tube center distance (mm), tank volume(kg), collector area (m^2^), angle (°) and final temperature (°C) are[1600, 2200], [5, 64], [60,151], [70.433, 403.332], [1.27, 8.24], [30,85] and [46,68], respectively. More detailed descriptions about the design of *WaterHeater*are covered in S1 File.

### Comparison with Conventional Methods

**Fig 7:**
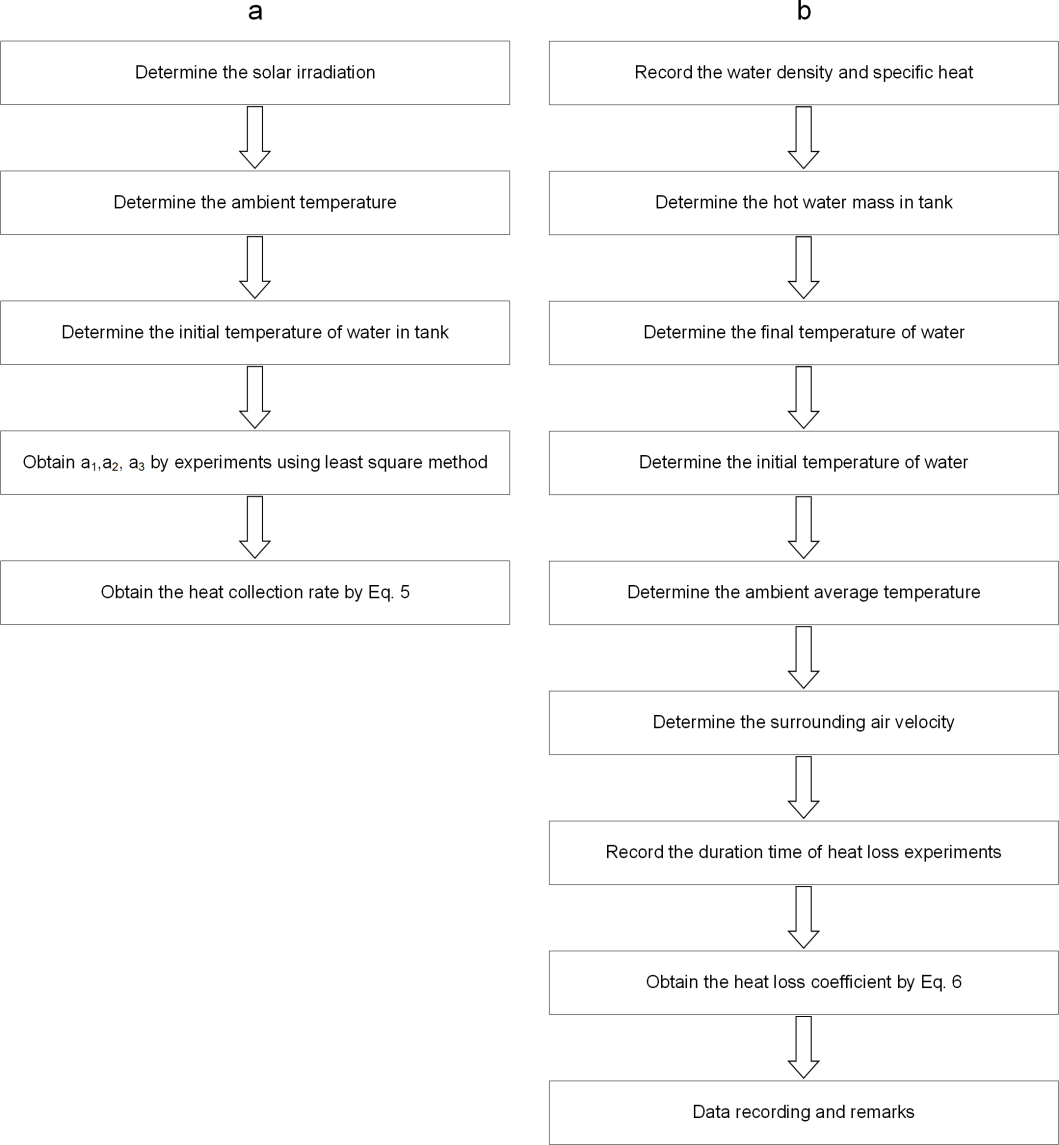
Flow chart of the conventional method for determining. (a) Heat collection. (b) Heat loss coefficient.

Using conventional methods for determination of heat collection rate and heat loss coefficient[31], a series of steps using detection devices needs to be completed (Fig 7). The pipes of water-in-glass evacuated tube solar water heaters need to be dismantled at first. However, for the determination tothe in service heaters, the disassembly of the pipes of heater is highly inconvenient and will cause damage to the instruments. Furthermore, the whole determination process requires at least 15 days. After a complicated determination process, the heat collection rate and heat loss coefficient canbe obtained by empirical equations [Eqs (5) and (6)], which is from ISO9459-2[32].

$$q_{s}=\frac{a_{1}H+a_{2}(t_{a}-t_{i})+a_{3}}{S}$$ (5)

$$U_{s}=\frac{\rho_{w}C_{p,w}V_{s}}{V\Delta \tau }\text{ln}\left[\frac{t_{is}-t_{as(av)}}{t_{fs}-t_{as(av)}}\right]$$ (6)

Where *q*_*s*_ is the heat collection rate, *H* is the amount of solar radiation, *t*_*a*_ is the ambient temperature, *t*_*i*_is the initial temperature of water in tank, *S* is the area of tubes and *a*_*1*_, *a*_*2*_and *a*_*3*_ are the coefficients; *U*_*s*_ is the heat loss coefficient, *ρ*_*w*_ is the water density, *C*_*p*,*w*_ is the specific heat of water, *V*_*s*_ is the heat water mass in tank, *t*_*is*_ is the initial temperature of water, *t*_*fs*_ is the final temperature of water, *t*_*as(av)*_ is the ambient average temperature, *V* is the volume of water and *Δτ* is the duration time of heat loss coefficient experiments.

**Fig 8:**
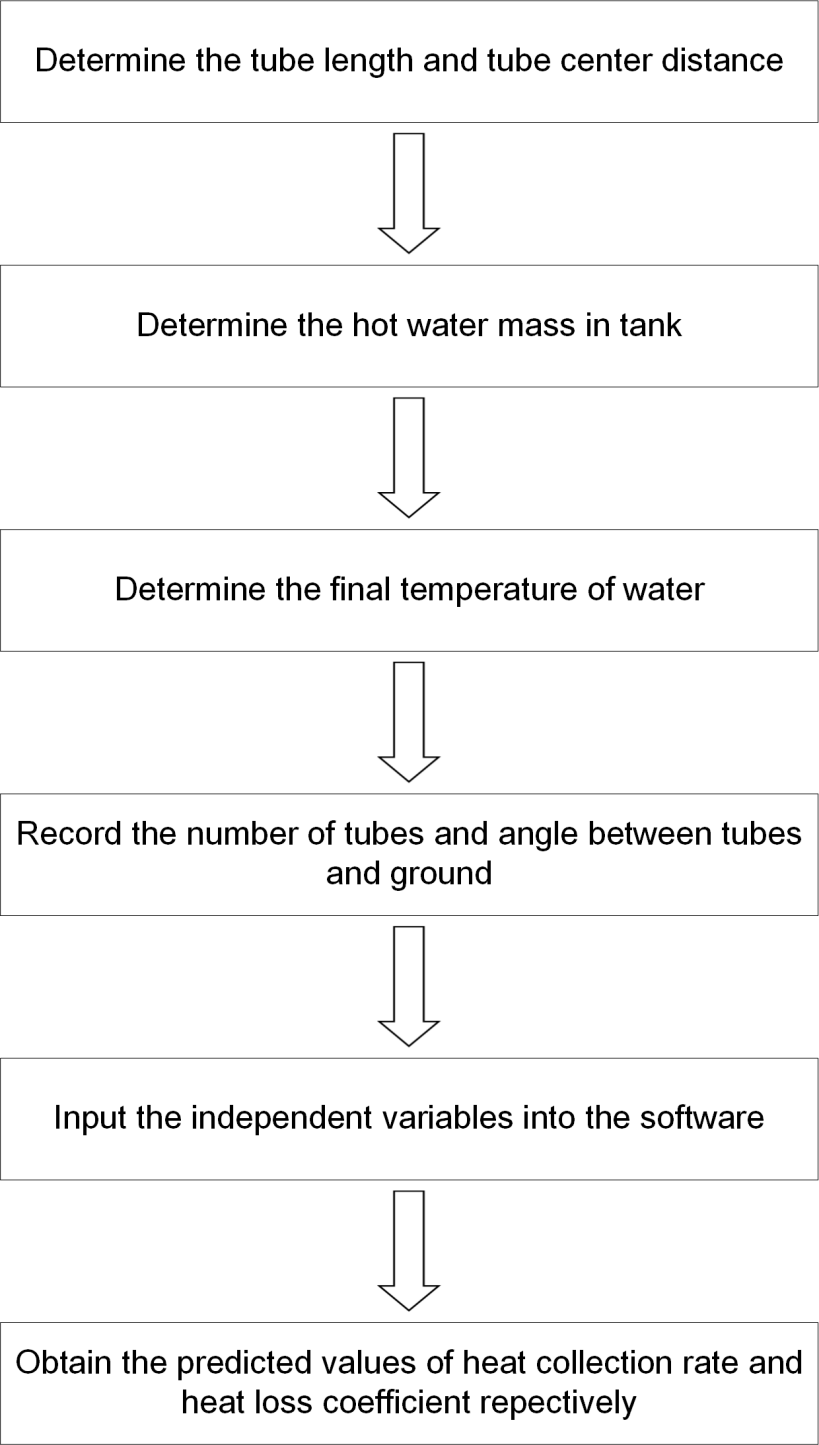
Flow chart of the novel method using "portable test instruments" combined with ANNs for determining heat collection rate and heat loss coefficient.

To optimize the determination method, herein, we propose a novel method to avoid the disassembly of the heaters, and meanwhile, largely shorten the testing time. The determination process using our novel method shows that the independent variables obtained by the "portable test instruments" can be inputted into the ANNs (Fig 8). The predicted results of heat collection rate and heat loss coefficient can be obtained precisely from the output of the ANNs. The use of "portable test instruments" is of great briefness and the combination of ANNs can greatly save time and give highly precise predicted results.

**Fig 9:**
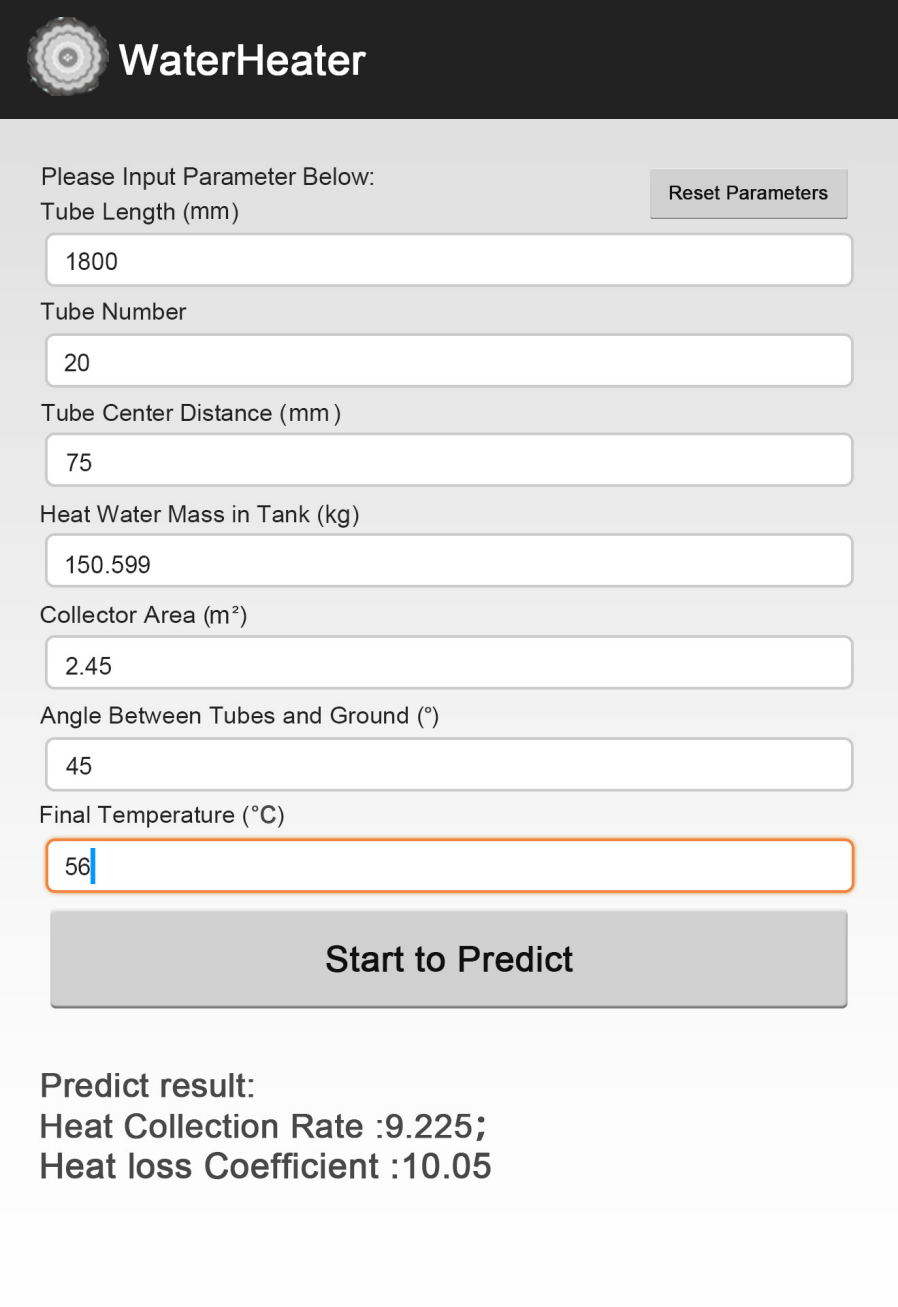
Results of an instance on Android platform.

**Fig 10:**
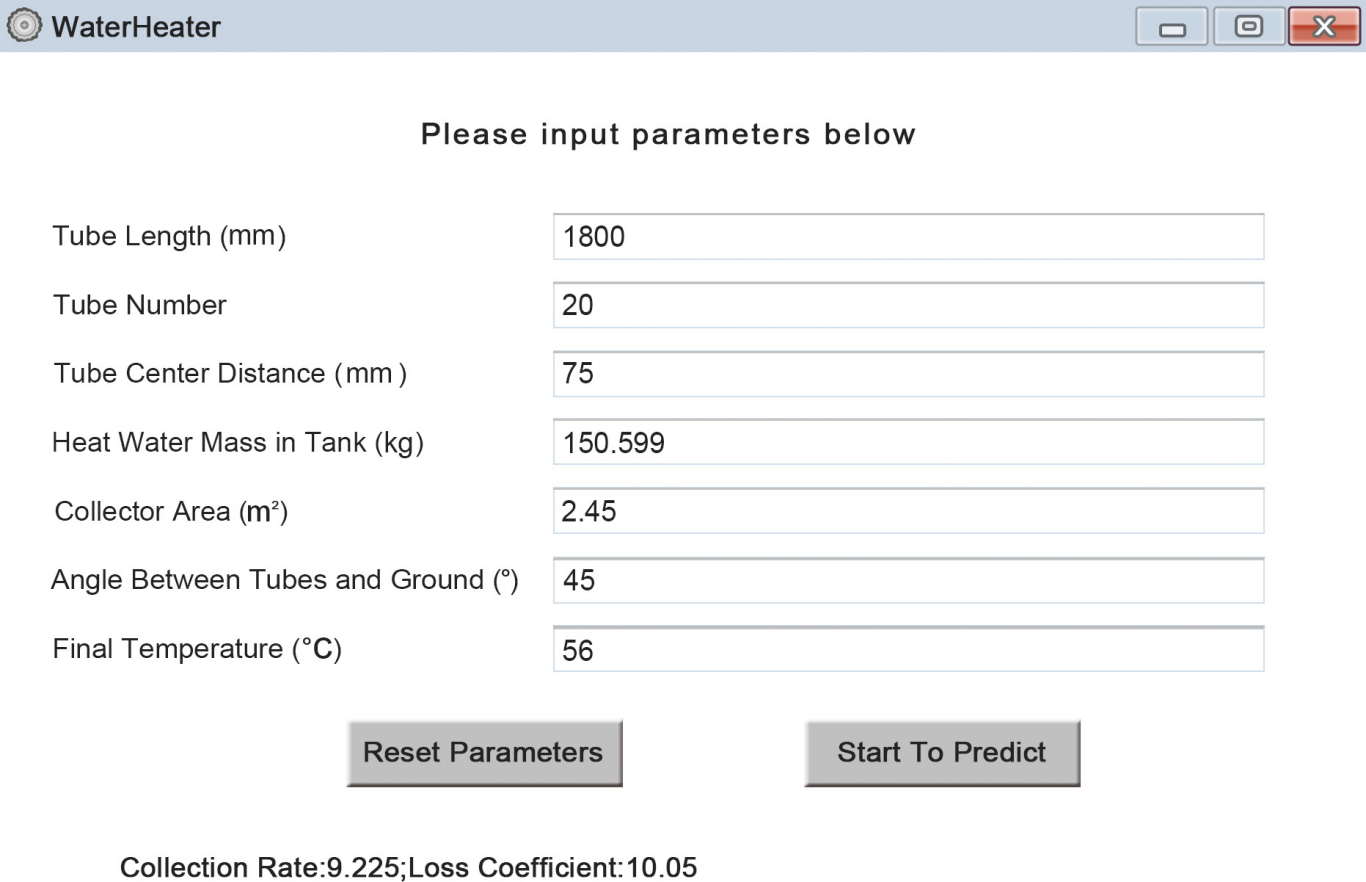
Results of an instance on PC platform.

Here, we present one case using both conventional method and the new one. According to the methods for independent and dependent variable determination in this study, the values of independent variables of our chosen water-in-glass evacuated tube solar water heater are 1800 mm, 20, 75 mm, 150.599 kg, 2.45 m^2^, 45°, 56°C respectively for tube length, number of tubes, tube center distance, tank volume, collector area, angle between tubes and ground and final temperature. With traditional method, the corresponding heat collection rate and heat loss coefficient are9.2 MJ/m^2^ and 10 [W/(Km^3^)] respectively, while with new method, the results are 9.225 MJ/m^2^ and 10.05[W/(Km^3^)]respectively. Figs 9 and 10 show the results on both Android platform and PC platform.

## Conclusion

*WaterHeater*, the ANNs-based software for predicting heat collection rate and heat loss coefficient of water-in-glass evacuated tube solar water heater is now developed. Results show that the BP models have high performances in predicting the heat collection rate and heat loss coefficient. The BP model with 200 epochs, 7 nodes, 0.9 learning rate, and 0.9 momentum has the best prediction performance for heat collection rate, with an RMS error 0.2049, while the BP model with 50 epochs, 1 hidden node, 0.1 learning rate, and 0.1 momentum has the best prediction performance for heat loss coefficient, with an RMS error 0.9246.In practical applications, the determination of heat collection rate and heat loss coefficient can be conducted outdoors using "portable test instruments". Data of independent variables obtained by "portable test instruments" can be inputted into the software and the precise predicted values of heat collection rate and heat loss coefficient can be obtained rapidly from the output of the models. Therefore, using our novel techniques, the determination of heat collection rate and heat loss coefficient is not to be performed in laboratories after being dismantled, which avoids the possible damage to the instruments and reduces manpower, experimental time and unnecessary operations. The software on both PC and Android platform can be currently downloaded from: http://t.cn/RLPKF08.

Furthermore, most of previous studies in the applications of ANNs to energy systems only proposed the methodology for model developments. However, lacking of details of programming and experimental data, other scientists and users usually found that it is difficult to repeat the same model process and use the method effectively. In this study, the development of the software, *WaterHeater*, based on the trained results of ANNs can avoid this common problem. Users can input the independent variables they acquired and the two coefficients can be acquired automatically without the acquisition of experimental data for training and the complex model development process of ANNs.

## Supporting Information

S1 File Detailed descriptions of the design of *WaterHeater*. This also includes flow chart and user interface of *WaterHeater*.
(DOCX)

S1 Table Results of prediction models for heat collection rate. (XLSX)

S2 Table Results of prediction models for heat loss coefficient. (XLSX)
