# Supplementary material for: Artificial Neural Networks-Based Software for Measuring Heat Collection Rate and Heat Loss Coefficient of Water-in-Glass Evacuated Tube Solar Water Heaters
Source: PLoS One. 2015 Dec 1;10(12):e0143624. doi: 10.1371/journal.pone.0143624 (PMC4666653; doi:10.1371/journal.pone.0143624)
Supplement: S1 File — This also includes flow chart and user interface of WaterHeater. (DOCX) [file pone.0143624.s001.docx]

# Software Design

Usability of an interface system is a measure of the amount of effort of a user must expend to learn its use and then use it to perform his or her routine in the workplace [1]. In this study, our goal is to satisfy users’ requirement for predicting heat collection rate and heat loss coefficient by a convenient way, leading us to develop the software, WaterHeater, with certain key features. Firstly, WaterHeater must have high usability, measured through ease of use, ease of learning and perceived gains in using it. Considering the platforms users might use, WaterHeater should be capable in varied environments [2].

In order to apply the system to varied environments, both personal computer (PC) and mobile platform were tested. Java language, which is known for Java Object Oriented Programming [3], was used throughout the project so that the artificial neural networks (ANNs) module could be easily packed into different platforms. Android operating system, which has been widely used nowadays, was chosen as a representative of mobile platforms. In our system, the minimum sdk version for android is 9 and target sdk version is 17. In terms of the PC platform, Java Runtime Environment (allowing executing a computer program compiled into [Java bytecode](https://en.wikipedia.org/wiki/Java_bytecode), designed by [Oracle Corporation](https://en.wikipedia.org/wiki/Oracle_Corporation)) has been embedded in the PC platform system, so that the system can be applied to different environments.

We used java language to preform ANNs for predicting function according to ANNs and back propagation algorithm and then encapsulated the function into a module, named class in Java, which can be packaged into both mobile and PC platforms [4]. The module was divided into two main components including reading normalized data, looking for the best bias and weight according to error. 815 groups of experimental data were firstly read into the module, and bias and weight were randomly set. One set of data was read into the input layer of the ANN each time. Values from the input layer were computed with bias and weight and transferred to the hidden layer. Hidden layer calculated values received using transfer function, computed with bias and weight and forwarded values to the output layer. After a series of ‘forward’ actions, ANNs output values were compared with actual output values and loss values were calculated with respects to all weights and bias in the network. Weight and bias were updated according to the loss values. The best weight and bias could be produced when the training with all 815 sets of data finished.

## Android platform

The whole Android software development was conducted in Android Developer Tools including Eclipse Platform (Android Developer Tools is a plugin for Eclipse that provides a suite of tools to help developers develop Android application quickly) [5]. Firstly, the graphical layout and main logical module of software were designed and coded, respectively. Then each component in the graphical layout was bundled with corresponding function in main logical module. ANNs predicting module was related to certain function in main logical module. When particular component in graphical layout was invoked, which sometimes involved pressing buttons, related function in main module would call ANNs predicting module. Then two ANNs instances including heat collection rate and heat loss coefficient started to train using the 815 sets of data in a proper order. After training, established ANNs models received seven independent values from input boxes and output the two dependent values, heat collection rate and heat loss coefficient, which were displayed in a panel component in the graphical layout.

## PC platform

We developed the PC-based software in Eclipse (an integrated development environment useful to develop applications). The user interface was designed with Java Graphical User Interface (GUI) which is a type of interface that allows users to interact with electronic devices through graphical icons and visual indicators [6]. Similar to the design in Android development, we added a particular event function on certain component in layout, named ‘control’ in GUI. When particular control was invoked, ANNs predicting module was woken up and two ANNs models including heat collection rate and heat loss coefficient were produced after training. Data from input boxes were imported into the established models and predicted results were displayed on the label in GUI layout.

# Flow Chart of WaterHeater

Fig 1 shows the flow chart of WaterHeater. The system firstly examined the validity of input independent variables. If invalid, a new set of input was required. Else, the training part of ANNs was prompted and the valid data was stored. Then the ANNs training part starts to work. The gradient descent algorithm was used in ANNs model, which performed a feed-forward pass, computing the activations for layers and then updated the training parameters according to the errors between ANNs output and actual output in the data sets [7]. After training, the best bias and weight were used to test the data stored before. Finally, output results were displayed on the panel.


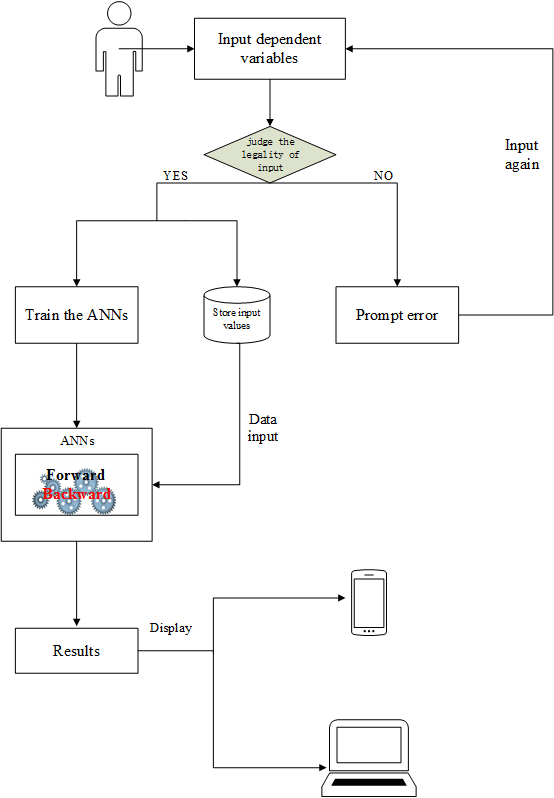


**Fig 1. The flow chart of WaterHeater**

# User Interface of WaterHeater

The design of WaterHeater has two major components in its architecture. One component deals with ANNs training and testing. The second component is the coordinated set of panels in which the end user navigates back and forth. ADT provides a complete user interface design environment for Android platform. While in terms of personal computer platform, GUI is adopted to design the user interface. Two platforms share many similarities in the work flow of user interface here:

As the first step, a user will be asked to input certain parameters, including tube length, number of tubes, tube center distance, heat water mass in tank, collector area, angle between tubes and ground and final temperature. Then the system will wait for instruments from users.

When users press ‘start to predict’ button, the core ANNs program begins. A single thread is started to train the neural net using the 815 data sets. Once the training ends, the system will pass data from input box to the established ANNs model and output the results, which will be displayed under the buttons.

If users press ‘Reset’ button, all input values and output values will disappear. Now, users can start a new instance for predicting.

# Reference

1. Ergonomics of human–system interaction—usability methods supporting human-centered design, vol. ISO 16982. (2002) International Organization for Standardization (ISO). 42 p.
2. FletcherD, GossE. (1993)Forecasting with neural networks: an application using bankruptcy data. Inform Manage-Amster. 24(3):159-167.
3. https://en.wikipedia.org/wiki/Java_(programming_language)
4. Heaton J. (2008) Introduction to neural networks with Java, Heaton Research, Inc.172p.
5. Meier R. Professional Android 4 application development, John Wiley & Sons, 2012.
6. AhoP, MenzN, RatyT, SchieferdeckerI. (2011) Automated Java GUI modeling for model-based testing purposes, In Information Technology: New Generations (ITNG), Eighth International Conference on. IEEE. 268-273.
7. HeinemanGT, CouncillWT. (2001) Component-based software engineering. Putting the Pieces Together Addison-Westley.
